# Protein phosphatase PPM1B inhibits DYRK1A kinase through dephosphorylation of pS258 and reduces toxic tau aggregation

**DOI:** 10.1074/jbc.RA120.015574

**Published:** 2021-01-08

**Authors:** Ye Hyung Lee, Eunju Im, Minju Hyun, Joongkyu Park, Kwang Chul Chung

**Affiliations:** 1 Department of Systems Biology, College of Life Science and Biotechnology, Yonsei University, Seoul, Korea; 2 Department of Pharmacology, School of Medicine, Wayne State University, Detroit, Michigan, USA

**Keywords:** DYRK1A, PPM1B, kinase, phosphatase, tau, Alzheimer’s disease, AD, Alzheimer’s disease, DAPI, 4′,6-diamidino-2-phenylindole, DMEM, Dulbecco’s modified eagle medium, DYRK1A, dual-specificity tyrosine-(Y)-phosphorylation regulated kinases, ECL, enhanced chemiluminescence, FBS, fetal bovine serum, HA, hemagglutinin, HEK293, human embryonic kidney 293, HRP, horseradish peroxidase, PPM, Metal-dependent protein phosphatase, TBST, Tris-buffered saline with Tween 20

## Abstract

Down syndrome (DS) is mainly caused by an extra copy of chromosome 21 (trisomy 21), and patients display a variety of developmental symptoms, including characteristic facial features, physical growth delay, intellectual disability, and neurodegeneration (*i.e.*, Alzheimer’s disease; AD). One of the pathological hallmarks of AD is insoluble deposits of neurofibrillary tangles (NFTs) that consist of hyperphosphorylated tau. The human *DYRK1A* gene is mapped to chromosome 21, and the protein is associated with the formation of inclusion bodies in AD. For example, DYRK1A directly phosphorylates multiple serine and threonine residues of tau, including Thr212. However, the mechanism underpinning DYRK1A involvement in Trisomy 21-related pathological tau aggregation remains unknown. Here, we explored a novel regulatory mechanism of DYRK1A and subsequent tau pathology through a phosphatase. Using LC-MS/MS technology, we analyzed multiple DYRK1A-binding proteins, including PPM1B, a member of the PP2C family of Ser/Thr protein phosphatases, in HEK293 cells. We found that PPM1B dephosphorylates DYRK1A at Ser258, contributing to the inhibition of DYRK1A activity. Moreover, PPM1B-mediated dephosphorylation of DYRK1A reduced tau phosphorylation at Thr212, leading to inhibition of toxic tau oligomerization and aggregation. In conclusion, our study demonstrates that DYRK1A autophosphorylates Ser258, the dephosphorylation target of PPM1B, and PPM1B negatively regulates DYRK1A activity. This finding also suggests that PPM1B reduces the toxic formation of phospho-tau protein *via* DYRK1A modulation, possibly providing a novel cellular protective mechanism to regulate toxic tau-mediated neuropathology in AD of DS.

Down syndrome (DS) is caused by the presence of an extra whole or partial copy of human chromosome 21 (trisomy 21) (1). DS patients show a variety of developmental symptoms, such as congenital heart defects, immune and endocrine malfunction, intellectual disability, and early-onset Alzheimer’s disease (AD) (1). Both DS and AD share common pathological hallmarks, including amyloid plaques and neurofibrillary tangles (NFTs) consisting of amyloid-β (Aβ) and hyperphosphorylated tau, respectively (2).

In healthy neurons, tau binds to and stabilizes microtubules that are critical for proper axonal outgrowth and synaptic plasticity (3). Neuropathological features within the affected brain regions in AD and Parkinson's disease (PD) are associated with the defective tau proteins that no longer stabilize microtubules (4).

DYRK1A belongs to the conserved protein kinase family called as dual-specificity tyrosine-(Y)-phosphorylation-regulated kinases (DYRKs), which includes DYRK1A, DYRK1B, DYRK2, DYRK3, and DYRK4 (5). DYRK1A is the only member located on chromosome 21 and contains multiple protein domains (6). Even though DYRK1A has a nuclear localization signal and 13-consecutive His-repeat domain (7), its substrates include both nuclear and cytosolic proteins, such as transcription factors, endocytosis and synaptic vesicle-recycling proteins, and cytosolic proteins, implying that DYRK1A participates in various biological responses (8).

Moreover, DYRK1A phosphorylates several neurodegenerative disease (NDD)-associated proteins, including tau, amyloid precursor protein (APP), and α-synuclein (9), affecting the formation of toxic protein inclusions in AD and PD (9, 10). For example, DYRK1A contributes to the hyperphosphorylation of tau and the formation of hyperphosphorylated tau aggregates. DYRK1A directly phosphorylates multiple serine (S199, S202, S396, S400, S404, and S422) and threonine (T181, T205, T212, T217, and T231) residues of tau (11). As excessive or abnormal phosphorylation of tau is believed to promote the transformation of normal adult tau into paired-helical-filament tau and NFTs, this so-called “tau hypothesis” states that the stage of disease determines NFT phosphorylation (12).

Members of the metal-dependent protein phosphatase (PPM) family are mostly monomeric Ser/Thr phosphatases and require the presence of Mg^2+^ or Mn^2+^ for catalytic activity (13). PPM1A is located in both the cytoplasm and nucleus and negatively regulates the activities of MAP kinases and MAP kinase kinases, inhibiting the activation of p38 and JNK kinase cascades induced by environmental stresses (14). PPM1B primarily present in the cytoplasm dephosphorylates cyclin-dependent kinases (CDKs), causing cell growth arrest or cell death (15). PPM1B also acts as IKKβ-phosphatase to terminate TNFα-induced IKKβ and NF-kB activation (13).

As the name implies, DYRK1A kinase possesses dual specificity due to its capability to autophosphorylate three amino acids (S97, T321, and S529) as well as to phosphorylate its substrates at serine or threonine residues (16). Whereas a number of DYRK1A substrates have been reported up to now, the precise molecular mechanism underlying regulation of its kinase activity and/or modulators involved remains to be elucidated. In this report, we have employed an electrospray ionization–mass spectrometry technique to identify novel binding proteins of DYRK1A in mammalian cells by screening a FLAG-tagged DYRK1A protein complex. As a result, we found that PPM1B binds to DYRK1A and acts a novel modulator of DYRK1A activity. The present work also demonstrates that DYRK1A autophosphorylates residue S258, and PPM1B dephosphorylates this pSer258, inhibiting the catalytic activity of DYRK1A and consequently reducing tau phosphorylation, its oligomerization, and toxic aggregation.

## Results

### DYRK1A binds to protein phosphatase PPM1B in mammalian cells

**Figure 1:**
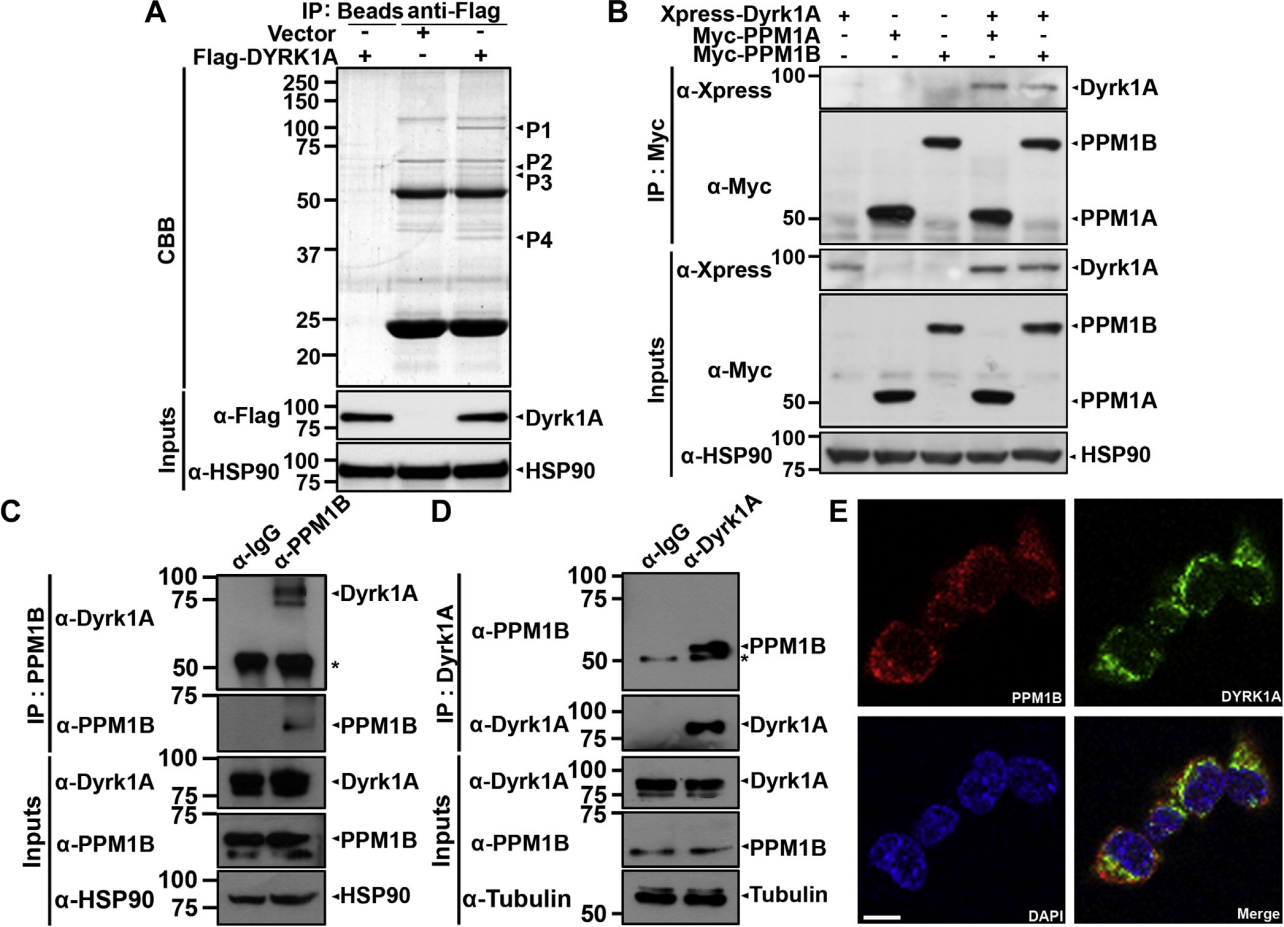
**DYRK1A binds to PPM1B in mammalian cells.***A*, HEK 293 cells overexpressing either FLAG-tagged DYRK1A (Flag-DYRK1A) or parental vector (Vector) were immunoprecipitated with anti-FLAG antibody, separated by SDS-PAGE, and the resolved proteins were visualized by Coomassie Brilliant Blue (CBB) G250 staining. As a negative control, cell lysates were immunoprecipitated with preimmune IgG (beads). The four indicated bands (P1‒P4) were then in-gel digested and analyzed by LC-MS/MS. The proper expression of stably transfected proteins in cell lysates was identified with immunoblot analysis using anti-Flag antibody. Hsp90 served as a loading control. *B*, SH-SY5Y cells were transfected for 24 h with plasmid encoding Xpress-DYRK1A, Myc-PPM1A, or Myc-PPM1B alone or in combination. Cell lysates were immunoprecipitated with anti-Myc antibody, followed by immunoblotting with anti-Xpress antibody. The proper expression of transiently transfected proteins in cell lysates was identified with immunoblot analysis using anti-Xpress or anti-Myc antibodies. Hsp90 served as a loading control. *C* and *D*, HEK293 cells lysates were immunoprecipitated with anti-PPM1B (*C*) or anti-DYRK1A antibody (*D*), followed by immunoblotting with anti-DYRK1A (*C*) or anti-PPM1B antibody (*D*). As a control, cell lysates were immunoprecipitated with preimmune IgG (IgG). *Asterisk* indicates IgG heavy chain. Hsp90 (*C*) or tubulin (*D*) served as a loading control, respectively. *E*, representative confocal images of immunostaining of HEK293 cells expressing both HA-DYRK1A (*green*) and Myc-PPM1B (*red*) are shown. Nuclei were counterstained with DAPI (*blue*). Scale bar = 10 μm.

To reveal the molecular mechanism underlying regulation of DYRK1A activity, the novel DYRK1A-interacting proteins were firstly analyzed in HEK 293 cells stably overexpressing epitope FLAG-tagged wild-type DYRK1A (Fig. 1*A*). Analyses of anti-DYRK1A complexes using the LC-MS/MS technique identified a previously known DYRK1A-binding protein, DDB1- and CUL4-associated factor 7 (denoted as P4; also known as WD-repeat domain protein 68 or WDR78) and its novel binding partner, Mg^2+^/Mn^2+^-dependent protein phosphatase 1B (PPM1B; marked as P3; Fig. 1*A*). In addition, P1 band was identified as the bait protein DYRK1A and P2 was an unnamed protein product (Fig. 1*A*).

We next examined whether DYRK1A binds to two PPM family proteins (PPM1A and PPM1B) in other mammalian cells, such as dopaminergic neuroblastoma SH-SY5Y cells. After transiently transfecting cells with Xpress-DYRK1A, Myc-PPM1A, and/or Myc-PPM1B, cell lysates were immunoprecipitated with anti-Myc antibody. Immunoblot analysis of anti-Myc immunocomplexes with anti-Xpress antibody revealed that DYRK1A binds to both PPM1A and PPM1B (Fig. 1*B*). Next, immunoblotting of anti-PPM1B immunocomplexes with anti-DYRK1A antibody demonstrated that endogenous DYRK1A interacts with endogenous PPM1B in HEK293 cells (Fig. 1*C*). When the coimmunoprecipitation assays were performed in a reverse order, we observed the same result (Fig. 1*D*). To determine whether DYRK1A colocalizes with PPM1B, HEK293 cells were transfected with Myc-PPM1B and HA-DYRK1A. Immunostaining of cells with anti-Myc (red) and anti-HA (green) antibodies revealed both proteins are commonly expressed in the cytosol (Fig. 1*E*).

These data suggest that the DYRK1A interacts with PPM1B in mammalian cells.

### PPM1B dephosphorylates DYRK1A at pSer residue(s)

**Figure 2:**
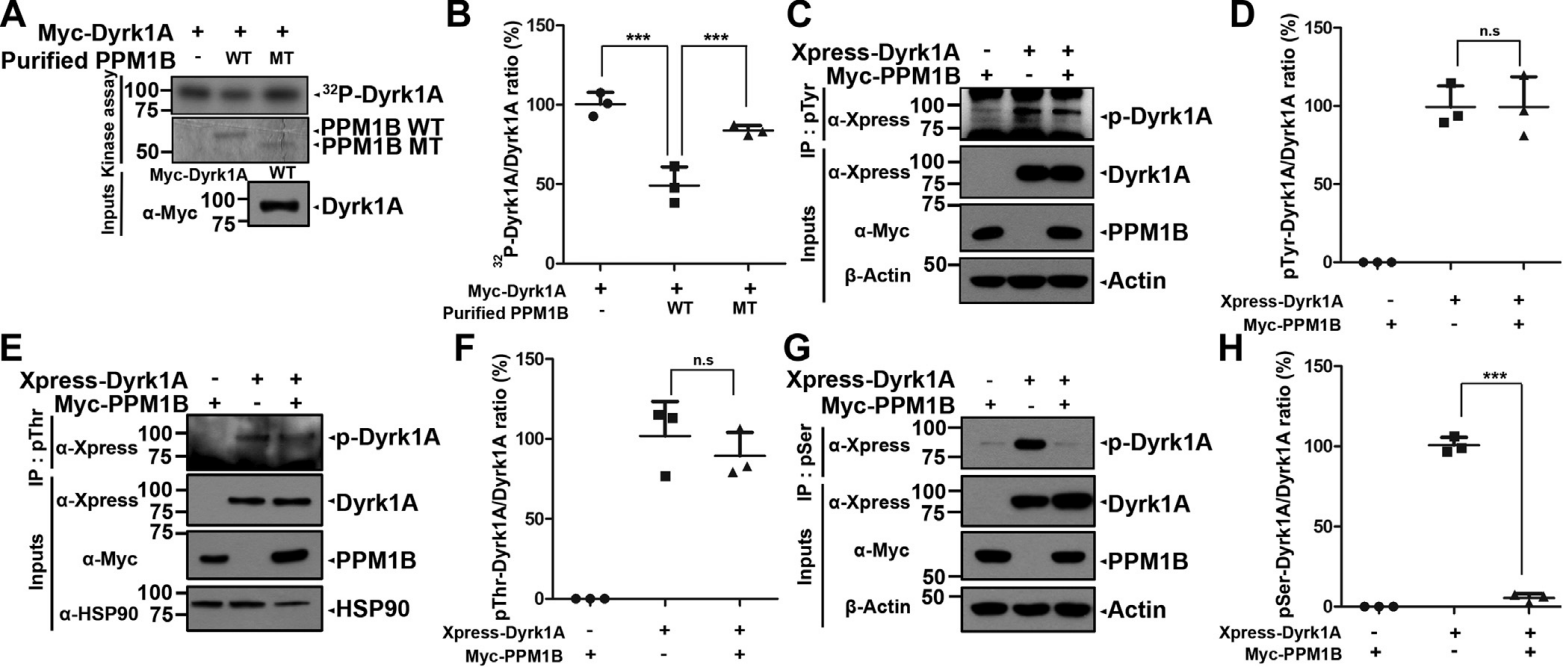
**PPM1B dephosphorylates DYRK1A at Ser residue(s).***A* and *B*, after HEK293 cells were transfected for 24 h with plasmid encoding Myc-DYRK1A-WT, three equal cell lysates including ∼1000 μg of protein were immunoprecipitated with anti-Myc antibody. The samples were equally incubated for 30 min at 30 °C with kinase buffer and [γ-^32^P]ATP to induce DYRK1A autophosphorylation. Where specified, the reaction products were then left untreated or mixed with bacterially expressed wild-type PPM1B (WT) or its phosphatase-inactive mutant (MT), incubated for 30 min at 30 °C, resolved by SDS-PAGE, and analyzed by autoradiography. The recombinant PPM1B bands were stained with CBB. Proper expression of transiently expressed DYRK1A in cell extracts was verified by western blotting with anti-Myc antibody (Input). The ratio of ^32^P-DYRK1A to DYRK1A in the graph was calculated by dividing the value of ^32^P-DYRK1A to presumed equal amount of DYRK1A in each sample. All graph data represent the mean ± SD of three independent experiments (∗∗∗*p* < 0.001). Statistical analyses were performed using the IBM SPSS Statistics software (version 23.0). *C*‒*H*, HEK293 cells were transfected for 24 h with plasmid encoding Xpress-DYRK1A or Myc-PPM1B alone or in combination. Cell lysates were immunoprecipitated with anti-pTyr (*C* and *D*), anti-pThr (*E* and *F*), or anti-pSer antibodies (*G* and *H*), followed by immunoblotting with anti-Xpress or anti-Myc antibodies. The proper expression of transiently transfected proteins in cell lysates was identified with immunoblot analysis using anti-Xpress or anti-Myc antibodies. β-Actin (*C* and *G*) and Hsp90 (*E*) served as a loading control. The ratios of pTyr-DYRK1A, pThr-DYRK1A, and pSer-DYRK1A to DYRK1A are shown in (*D*, *F*, and *H*), respectively. All graph data represent the mean ± SD of three independent experiments (∗∗∗*p* < 0.001; n.s., not significant).

To gain further insight as to how DYRK1A and PPM1B are linked, we addressed whether DYRK1A directly phosphorylates PPM1B and/or whether PPM1B dephosphorylates DYRK1A. To do this, we employed *in vitro* kinase and phosphatase assays (Fig. 2, *A* and *B*). Wild-type PPM1B (WT) protein and its phosphatase-inactive mutant having the substitution R179G (PPM1B-MT) were expressed in *Escherichia coli* and purified. For DYRK1A, HEK293 cells were transfected with Myc-DYRK1A, and the anti-Myc immunocomplexes were prepared. After anti-Myc-DYRK1A immunoprecipitates were incubated with [γ-^32^P]ATP alone or with either bacterially purified recombinant PPM1B-WT or PPM1B-MT, *in vitro* assays for protein phosphorylation and dephosphorylation revealed that DYRK1A efficiently autophosphorylates in the absence of PPM1B (Fig. 2, *A* and *B*). However, PPM1B was not measurably phosphorylated by DYRK1A. Interestingly, the amount of autophosphorylated DYRK1A was markedly reduced in the presence of wild-type PPM1B, whereas this effect was not seen with PPM1B-MT (Fig. 2, *A* and *B*). These results suggest that PPM1B dephosphorylates phospho-DYRK1A.

As DYRK1A has dual substrate specificity, DYRK1A autophosphorylates on Ser/Thr as well as Tyr residues for self-activation (5, 9). Based on these findings, we tested whether PPM1B could dephosphorylate DYRK1A at either Tyr or Ser/Thr residues. Cells were transfected with Xpress-DYRK1A and/or Myc-PPM1B, and cell lysates were immunoprecipitated with anti-pTyr, anti-pThr, or anti-pSer antibodies (Fig. 2, *C*–*H*). Immunoblotting of the samples with anti-Xpress antibody revealed that the amount of phosphorylated DYRK1A at Tyr or Thr residue was not reduced with PPM1B (Fig. 2, *C*–*F*). However, cotransfection of PPM1B and DYRK1A greatly reduced the level of phosphorylated DYRK1A at Ser residue(s), compared with the sample transfected with DYRK1A alone (Fig. 2, *G* and *H*).

Taken together, these results indicate that PPM1B dephosphorylates DYRK1A at the serine residue(s), but not tyrosine or threonine site.

### PPM1B has no effect on DYRK1A at residues pS310 or pS529

After analyzing the Phosphosite Database (http://www.phosphosite.org), two novel Ser sites within DYRK1A were predicted to serve as putative phosphorylation targets of PPM1B: Ser-310 and -324, respectively. X-ray analysis of DYRK1A protein structure indicated that the S310 is located close to the previously reported autophosphorylation site Y321 and facing outward (17). However, the other S324 is presumed not to be at the surface, so is not suitable for direct modification. Based on this speculation and the previous report that DYRK1A autophosphorylation on Ser529 (equivalent to the Ser520 of human 754-amino acid isoform) modulates its kinase activity *via* 14-3-3 binding (18), we firstly postulated that the potential Ser phosphorylation target(s) of PPM1B could be S310 or S529 and investigated the validity of the hypothesis.

**Figure 3:**
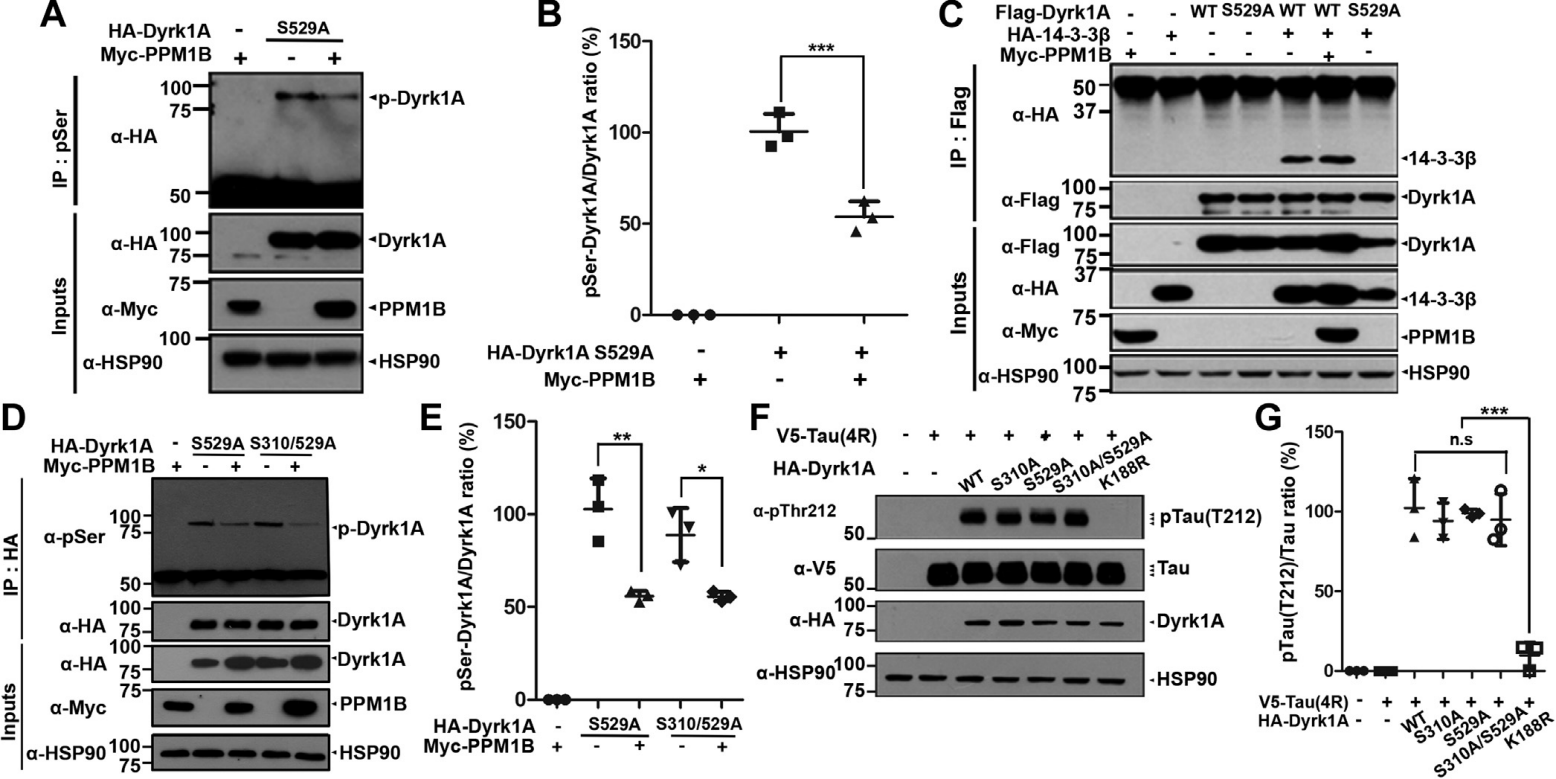
**PPM1B has no effect on phospho-DYRK1A at residues S310 or S529.***A* and *B*, HEK293 cells were transfected for 24 h with plasmids encoding HA-DYRK1A-S529A and/or Myc-PPM1B. Cell lysates were immunoprecipitated with anti-p-Ser antibody, followed by immunoblotting with anti-HA antibody. The proper expression of transiently transfected proteins in cell lysates was identified with immunoblot analysis using anti-HA or anti-Myc antibodies. Hsp90 served as a loading control. The ratio of pSer-DYRK1A to DYRK1A is shown in (*B*). All graph data represent the mean ± SD of three independent experiments (∗∗∗*p* < 0.001). *C*, HEK293 cells were transfected for 24 h with plasmids encoding FLAG-DYRK1A-WT, FLAG-DYRK1A-S529A, HA-14-3-3β, or Myc-PPM1B alone or in combination. Cell lysates were immunoprecipitated with anti-FLAG antibody, followed by immunoblotting with anti-HA antibody. The proper expression of transiently transfected proteins in cell lysates was identified with immunoblot analysis using anti-Flag, anti-HA, or anti-Myc antibodies. Hsp90 served as a loading control. *D*, HEK293 cells were transfected for 24 h with plasmids encoding HA-DYRK1A-S529A, HA-DYRK1A-S310/529A, or Myc-PPM1B alone or in combination. Cell lysates were immunoprecipitated with anti-HA antibody, followed by immunoblotting with anti-phospho-serine antibody. The proper expression of transiently transfected proteins in cell lysates was identified with immunoblot analysis using anti-HA or anti-Myc antibodies. *E*, all graph data represent the mean ± SD of three independent experiments (∗*p* < 0.05; ∗∗*p* < 0.01). *F*, HEK293 cells were transfected for 24 h with plasmids encoding V5-Tau or HA-DYRK1A-WT, or HA-DYRK1A-S310A, HA-DYRK1A-S529A, HA-DYRK1A-S310/529A, or HA-DYRK1A-K188R (kinase-inactive mutant of DYRK1A) alone or in combination. Cell lysates were immunoblotted with anti-pTau antibody (T212). The proper expression of transiently transfected proteins in cell lysates was identified with immunoblot analysis using anti-V5 or anti-HA antibodies. Hsp90 served as a loading control. *G*, all graph data represent the mean ± SD of three independent experiments (∗∗∗*p* < 0.001; n.s., not significant).

After HEK293 cells were transfected with HA-tagged DYRK1A-S529A or Myc-tagged PPM1B alone or in combination, cell lysates were immunoprecipitated with anti-pSer antibody. Immunoblot analysis of the immunocomplex with anti-HA antibody revealed that the phosphorylation status of DYRK1A-S529A was repressed to ∼50% by PPM1B, comparable with greatly reduced phosphorylation of wild-type DYRK1A (Fig. 3, *A* and *B*). According to the previous report, phosphorylation of DYRK1A at S529 also increases its binding affinity for 14-3-3β protein (18). On the basis of this finding, we further examined whether PPM1B changes the interaction between presumed autophosphorylated DYRK1A at S529 and 14-3-3β. As shown in Figure 3*C*, PPM1B had no effect on the binding of DYRK1A to 14-3-3β, verifying that PPM1B does not act at DYRK1A-S529. These results indicate that Ser529 is not the target site of PPM1B.

We next examined whether S310 could be a target site of PPM1B. Cells were transfected for 24 h with HA-DYRK1A-S529A, HA-DYRK1A-S310/529A, or Myc-PPM1B alone or in combination (Fig. 3, *D* and *E*). Lysates were then immunoprecipitated with anti-HA antibody. Immunoblotting of the samples with anti-pSer antibody revealed that DYRK1A-S310/S529A still displayed a considerable pSer band. In addition, DYRK1A-S310/529A displayed greatly reduced phosphorylation in the presence of PPM1B, similar to the pattern of DYRK1A-S529A. Moreover, comparing with the effect of DYRK1A-WT on tau phosphorylation, three DYRK1A point mutants (DYRK1A-S529A, DYRK1A-S310A, and DYRK1A-S529/310A) exhibit similar activity (Fig. 3, *F* and *G*). However, it was not seen with the kinase-inactive DYRK1A-K188R mutant, as expected.

Taken together, these results suggest that neither S310 nor S529 of DYRK1A is the target site of PPM1B.

### PPM1B selectively dephosphorylates DYRK1A at Ser258, which is the novel autophosphorylation site

**Figure 4:**
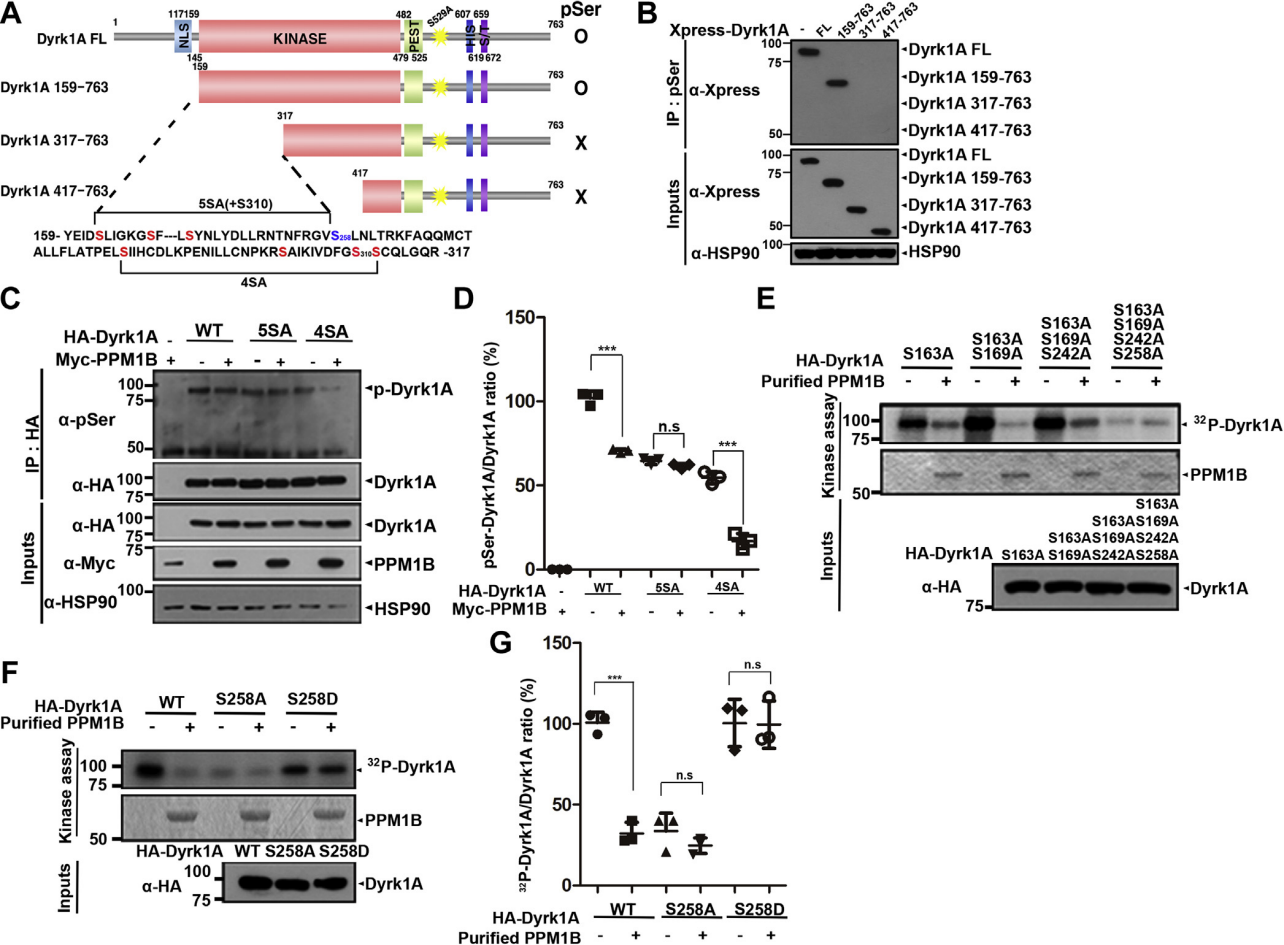
**PPM1B dephosphorylates DYRK1A at residue S258.***A*, schematic diagram of full-length DYRK1A (FL) and its truncated mutants, and the summary of co-IP experiments to detect the expression of pSer in those DYRK1A proteins. *B*, HEK293 cells were transfected for 24 h with plasmid encoding Xpress-DYRK1A-FL or its truncated mutants (*i.e.*, DYRK1A^159–763^, DYRK1A^317–763^, and DYRK1A^417–763^). Cell lysates were immunoprecipitated with anti-pSer antibody, followed by immunoblotting with anti-Xpress antibody. Proper expression of transiently expressed Dyrk1A was confirmed by immunoblotting with the anti-Xpress antibody. Hsp90 served as a loading control. *C*, HEK293 cells were transfected for 24 h with plasmid encoding HA-DYRK1A-WT, or its point mutants (HA-DYRK1A-5SA or HA-DYRK1A-4SA), or Myc-PPM1B alone or in combination. Cell lysates were immunoprecipitated with anti-HA antibody, followed by immunoblotting with anti-pSer antibody. The proper expression of transiently transfected proteins in cell lysates was identified with immunoblot analysis using anti-HA or anti-Myc antibodies. *D*, all graph data represent the mean ± SD of three independent experiments (∗∗∗*p* < 0.001; n.s., not significant). *E* and *F*, where indicated, HEK293 cells were transfected for 24 h with the plasmid encoding one of the HA-tagged DYRK1A mutants having the substitution at S163A, S163A + S169A, S163A + S169A + S242A, or S163A + S169A + S242A + S258A (*E*), HA-tagged wild-type DYRK1A or its DYRK1A-S258A mutant, or DYRK1A-S258D mutant (*F*). After cell lysis in lysis buffer, two equal cell lysates containing ∼1000 μg of protein were immunoprecipitated with anti-HA antibody. The samples were equally incubated for 30 min at 30 °C with kinase buffer and [γ-^32^P]ATP to induce DYRK1A autophosphorylation. The reaction samples were left untreated or mixed with bacterially expressed PPM1B-WT, incubated for 30 min at 30 °C, resolved by SDS-PAGE, and analyzed by autoradiography. Recombinant PPM1B-WT was stained with CBB, and DYRK1A expression in cell extracts was verified by western blotting with anti-HA IgG (Input). Proper expression of transiently expressed HA-tagged Dyrk1A-WT or its multiple mutants was confirmed by immunoblotting with the anti-HA antibody. *G*, the ratio of ^32^P-DYRK1A to DYRK1A in the graph was calculated by dividing the value of ^32^P-DYRK1A to the same amount of HA-DYRK1A-WT, HA-DYRK1A-S258A, and HA-DYRK1A-S258D in each sample. All graph data represent the mean ± SD of three independent experiments (∗∗∗*p* < 0.001; n.s., not significant).

To precisely locate the region of DYRK1A dephosphorylated by PPM1B, three DYRK1A deletion constructs, *i.e.*, DYRK1A^159–763^, DYRK1A^317–736^, and DYRK1A^417–736^ were generated (19). To exclude nonspecific and the masking effect of the phospho-DYRK1A signal at Ser529, the DYRK1A-S529A point mutant was utilized as the template for the production of these mutants. HEK293 cells were then transfected with wild-type DYRK1A or one of these deletion mutants, and lysates were immunoprecipitated with anti-pSer IgG. As shown in Figure 4, *A* and *B*, the DYRK1A^159–763^ fragment was still phosphorylated at Ser residue(s) in the absence of PPM1B to an extent similar to the full-length DYRK1A. However, DYRK1A^317–736^ and DYRK1A^417–736^ failed to be phosphorylated at Ser residue(s) (Fig. 4, *A* and *B*). These results indicated that the target serine residue is present between amino acid 159 and 316.

We next tried to identify the exact PPM1B-targeting site(s) on DYRK1A. As the DYRK1A^159–316^ region contains a total of eight serine residues, *i.e.*, S163, S169, S242, S258, S282, S301, S310, and S311, two DYRK1A mutants having the mutations of these sites were generated. The first DYRK1A-I-5SA mutant (DYRK1A-5SA) has five substitutions of Ser with Ala at S163, S169, S242, S258, and S310, and the second DYRK1A-II-4SA (DYRK1A-4SA) has four similar substitutions at S282, S301, S310, and S311 (Fig. 4*A*). After cells were transfected with one of these HA-tagged mutants alone or together with Myc-PPM1B, we measured the phosphorylation status of DYRK1A-5SA and -4SA mutants and compared with that of wild-type DYRK1A (Fig. 4, *C* and *D*). Immunoblotting of anti-HA immunocomplexes with anti-pSer IgG demonstrated that the phosphorylation levels of wild-type DYRK1A and DYRK1A-4SA were greatly reduced by PPM1B, but the amount of phospho-DYRK1A-5SA was unaffected by PPM1B (Fig. 4, *C* and *D*). These results suggest that PPM1B targets and dephosphorylates the serine residue(s) substituted within the DYRK1A-5SA mutant, which include S163, S169, S242, and S258.

To identify the exact PPM1B-targeted pSer residue(s) among these four residues, we next generated four DYRK1A mutants having the mutation of S163A or multiple mutations at S163A/S169A, S163A/S169A/S242A, or S163A/S169A/S242A/S258A and examined whether they were dephosphorylated. As shown in Figure 4*E*, three DYRK1A mutants having no S258A mutation were still efficiently dephosphorylated by PPM1B. However, comparing with other mutants, the phosphorylation level of the DYRK1A-S163A/S169A/S242A/S258A mutant was greatly reduced, and this level was not significantly altered in the presence of PPM1B (Fig. 4*E*). *In vitro* kinase assay further revealed that the S258A mutant displayed a reduction in DYRK1A phosphorylation by 75%, compared with wild-type DYRK1A (Fig. 4, *F* and *G*). In addition, the phosphorylation level of the DYRK1A-S258A mutant was not significantly affected by PPM1B. Moreover, the phosphorylation level of phospho-mimetic DYRK1A-S258D mutant was unaffected by PPM1B (Fig. 4, *F* and *G*).

Taken together, these data indicated that DYRK1A is constitutively autophosphorylated at Ser258, which serves as the target residue of PPM1B.

### PPM1B-mediated dephosphorylation of DYRK1A at Ser258 suppresses the kinase activity, leading to the reduction of tau phosphorylation at Thr212

Except for autophosphorylation at residues Tyr321 and Ser529, the identification of Ser258 as an autophosphorylation site and its modulatory effect on DYRK1A activity were not assessed at all. Therefore, we next examined whether and how the phosphorylation–dephosphorylation of DYRK1A at Ser258 affects its kinase activity. As described previously, DYRK1A phosphorylates a number of nuclear and cytosolic substrate proteins, including several NDD-associated targets (9). For example, DYRK1A-mediated phosphorylation of tau at Thr212 is known to contribute to toxic tau oligomerization and aggregation (11, 20). Based on these and to assess the regulatory effect of PPM1B on DYRK1A activity, we measured the extent of DYRK1A-mediated tau phosphorylation at Thr212.

**Figure 5:**
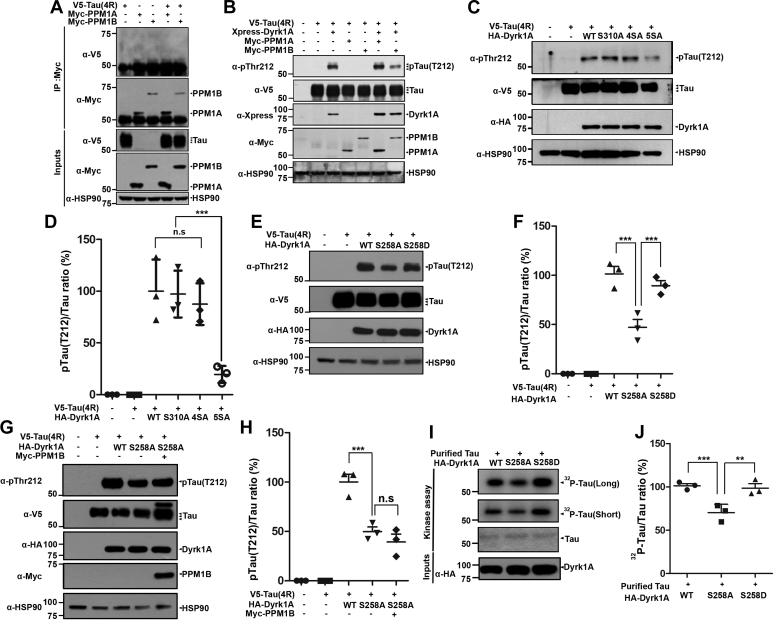
**PPM1B-mediated dephosphorylation of DYRK1A reduces tau phosphorylation at T212.***A*, HEK293 cells were transfected for 24 h with plasmid encoding V5-Tau, Myc-PPM1A, or Myc-PPM1B alone or in combination. Cell lysates were immunoprecipitated with anti-Myc antibody, followed by immunoblotting with anti-V5 antibody. The proper expression of transiently transfected proteins in cell lysates was identified with immunoblot analysis using anti-V5 or anti-Myc antibodies. Hsp90 served as a loading control. *B*, HEK293 cells were transfected for 24 h with plasmid encoding V5-Tau, Xpress-DYRK1A, Myc-PPM1A, or Myc-PPM1B alone or in combination. Cell lysates were immunoblotted with anti-pTau antibody (T212). The proper expression of transiently transfected proteins in cell lysates was identified with immunoblot analysis using anti-V5, anti-Xpress, or anti-Myc antibodies. *C*, HEK293 cells were transfected for 24 h with plasmid V5-Tau, HA-DYRK1A-WT, HA-DYRK1A-S310A, HA-DYRK1A-4SA, or HA-DYRK1A-5SA alone or in combination. Cell lysates were immunoblotted with anti-pTau antibody (T212). The proper expression of transiently transfected proteins in cell lysates was identified with immunoblot analysis using anti-V5 or anti-HA antibodies. *D*, All graph data represent the mean ± SD of three independent experiments (∗∗∗*p* < 0.001; n.s., not significant). *E*, HEK293 cells were transfected for 24 h with plasmid encoding V5-Tau, HA-DYRK1A-WT, HA-DYRK1A-S258A, or HA-DYRK1A-S258D alone or in combination. Cell lysates were immunoblotted with anti-pTau antibody (T212). The proper expression of transiently transfected proteins in cell lysates was identified with immunoblot analysis using anti-V5 or anti-HA antibodies. The pThr212-tau/tau ratio is shown in (*F*). All graph data represent the mean ± SD of three independent experiments (∗∗∗*p* < 0.001). *G*, HEK293 cells were transfected for 24 h with plasmid encoding V5-Tau, HA-DYRK1A WT, HA-DYRK1A S258A, or Myc-PPM1B alone or in combination. Cell lysates were immunoblotted with anti-pTau antibody (T212). The proper expression of transiently transfected proteins in cell lysates was identified with immunoblot analysis using anti-V5, anti-HA, or anti-Myc antibodies. *H*, all graph data represent the mean ± SD of three independent experiments (∗∗∗*p* < 0.001; n.s., not significant). *I*, HEK293 cells were transfected for 24 h with plasmid encoding HA-DYRK1A-WT, HA-DYRK1A-S258A, or HA-DYRK1A-S258D. Cell lysates were immunoprecipitated with anti-HA antibody, and the samples were incubated for 30 min with kinase buffer, recombinant tau, and [γ-^32^p]ATP. The reaction products were separated by SDS-PAGE and analyzed by autoradiography. Bacterial recombinant tau was stained with CBB. Proper expression of transiently expressed HA-tagged Dyrk1A-WT and its mutants was confirmed by immunoblotting with the anti-HA antibody. The ^32^P-tau/tau ratio is shown in (J). All graph data represent the mean ± SD of three independent experiments (∗∗*p* < 0.01; ∗∗∗*p* < 0.001).

Before determining the presumed regulatory effect of PPM1B on DYRK1A-mediated tau phosphorylation, we examined whether PPM1B is biochemically associated with tau as a control. HEK293 cells were transfected with plasmids encoding V5-Tau, Myc-PPM1B, and/or Myc-PPM1A, and cell lysates were immunoprecipitated with anti-Myc antibody. Immunoblot analysis of anti-Myc immunocomplexes with anti-V5 antibody revealed that tau does not bind to either PPM1B or PPM1A (Fig. 5*A*). We then determined how PPM1B and PPM1A affect the kinase activity of DYRK1A by measuring the change in DYRK1A-mediated tau phosphorylation at T212. After HEK293 cells were transfected with plasmids encoding Xpress-DYRK1A, V5-tau, Myc-PPM1A, or Myc-PPM1B alone or together, immunoblot analysis of cell lysates with anti-p-tau antibody (T212) showed that tau was not phosphorylated at Thr212 in the absence of DYRK1A (Fig. 5*B*). In addition, DYRK1A considerably promoted tau phosphorylation at T212, which was consistent with the previous report (9). Moreover, the level of tau phosphorylation was not affected by the presence of either PPM1B or PPM1A alone, as expected (Fig. 5*B*). However, the increase of tau phosphorylation in HEK293 cells overexpressing DYRK1A was significantly blocked by coexpression of PPM1B, whereas this effect was not observed with PPM1A (Fig. 5*B*). These results indicate that PPM1B decreases tau phosphorylation *via* DYRK1A activity. They also suggest that PPM1B inhibits DYRK1A activity.

To confirm this hypothesis, we tested the effect of several DYRK1A point mutants on tau phosphorylation (Fig. 5, *C* and *D*). Cells were transfected with V5-tau alone or together with HA-tagged DYRK1A-S310A, DYRK1A-4SA, or DYRK1A-5SA, and immunoblot analysis of cell lysates with anti-p-Tau IgG (T212) showed that wild-type DYRK1A and two other mutants (DYRK1A-S310A and DYRK1A-4SA) efficiently execute the phosphorylation of tau (Fig. 5, *C* and *D*). However, the DYRK1A-5SA mutant with the substitution S258A did not promote tau phosphorylation (Fig. 5, *C* and *D*). In addition, by using the same method employing three other DYRK1A point mutants, *i.e.*, the phosphorylation-resistant (DYRK1A-S258A) and phosphorylation-mimetic (DYRK1A-S258D) mutants, we measured their effect on tau phosphorylation. As shown in Figure 5*E*, the DYRK1A-S258A mutant failed to mediate tau phosphorylation at T212, compared with wild-type DYRK1A. However, it was rescued and restored to DYRK1A wild-type level by the DYRK1A-S258D mutant (Fig. 5, *E* and *F*). In addition, much reduced level of tau phosphorylation was not considerably affected by coexpression of PPM1B and DYRK1A-S258A (Fig. 5, *G* and *H*).

To verify that PPM1B-mediated dephosphorylation of DYRK1A at S258 reduces tau phosphorylation, HEK293 cells were transfected with plasmids expressing HA-DYRK1A-WT, HA-DYRK1A-S258A, or HA-DYRK1A-S258D, and cell lysates were immunoprecipitated with anti-HA antibody. Then, we performed an *in vitro* kinase assay with the anti-HA immunocomplex as the kinase, recombinant His-tagged tau as the substrate, and [γ-^32^p]ATP. Autoradiographic analysis of reaction products demonstrated that the DYRK1A-S258A mutant caused much less tau phosphorylation than DYRK1A-WT. On the contrary, phospho-mimetic mutant DYRK1A-S258D promoted tau phosphorylation to a level comparable with or slightly greater than DYRK1A-WT (Fig. 5, *I* and *J*).

Taken together, these data suggest that the autophosphorylation of DYRK1A at S258 appears to be essential for the kinase activity, but not enough by itself to enhance its activity. In addition, dephosphorylation of DYRK1A-S258 by PPM1B decreases its kinase activity, subsequently downregulating tau phosphorylation at T212.

### PPM1B reduces tau oligomerization and aggregation *via* negative regulation of DYRK1A

**Figure 6:**
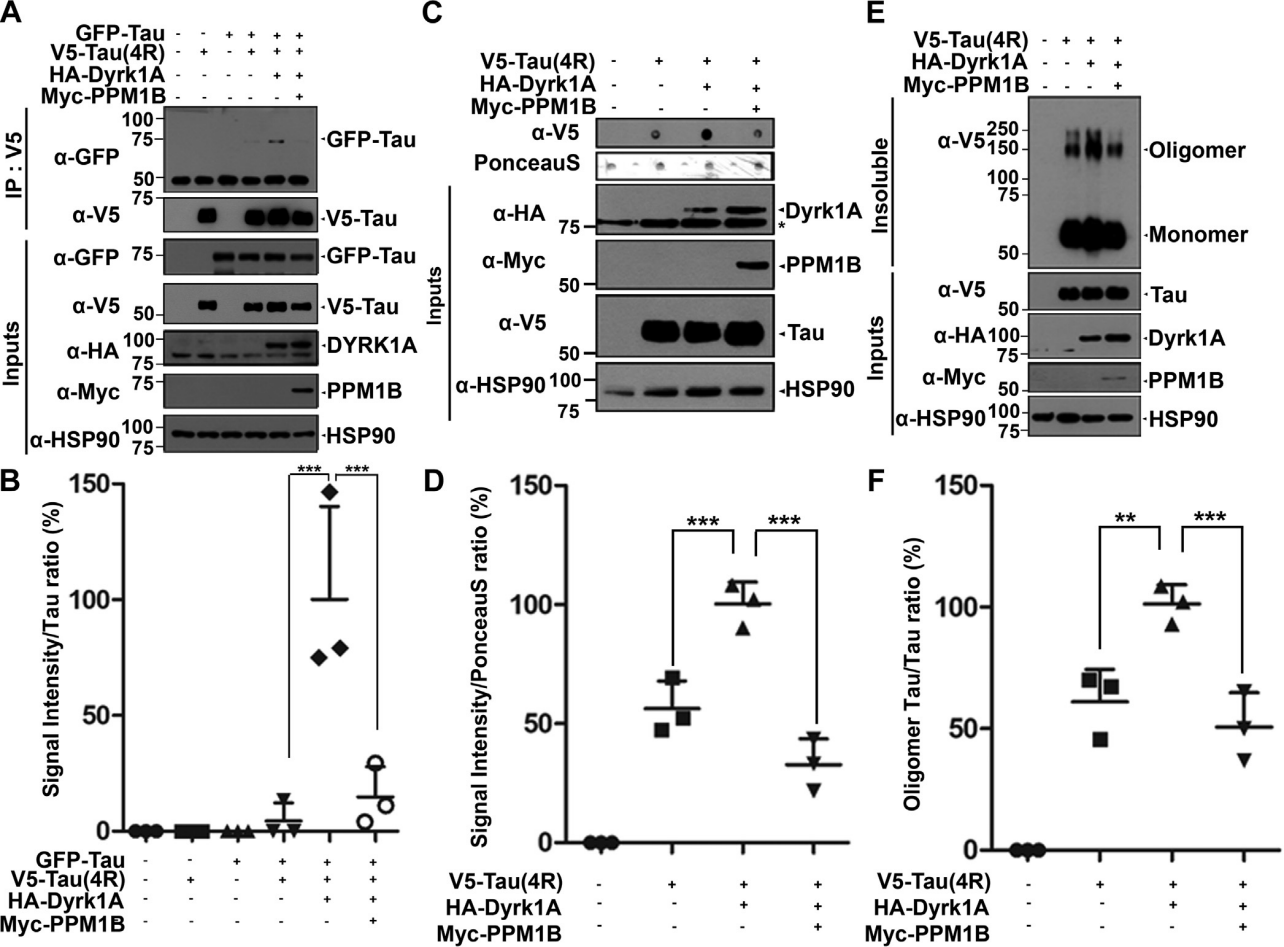
**PPM1B reduces the oligomerization and aggregation of pTau.***A*, HEK293 cells were transfected for 24 h with plasmid encoding GFP-Tau, V5-Tau, HA-DYRK1A, or Myc-PPM1B alone or in combination. Cell lysates were immunoprecipitated with anti-V5 antibody, followed by immunoblotting with anti-GFP antibody. The proper expression of transiently transfected proteins in cell lysates was identified with immunoblot analysis using anti-GFP, anti-V5, anti-HA or anti-Myc antibodies. Hsp90 served as a loading control. *B*, all data represent the mean ± SD of three independent experiments (∗∗∗*p* < 0.001). *C*, HEK293 cells were transfected for 48 h with plasmid encoding V5-Tau, HA-DYRK1A, or Myc-PPM1B alone or in combination. Cell lysates were analyzed by western blotting (Inputs), and the extent of tau oligomerization was analyzed by filter trap assay. The blots of the filter trap assay were subjected to either immunoblotting with anti-V5 antibody or Ponceau S staining. The proper expression of transiently transfected proteins in cell lysates was identified with immunoblot analysis using anti-V5, anti-HA or anti-Myc antibodies. Asterisk indicates a nonspecific band. *D*, all data represent the mean ± SD of three independent experiments (∗∗∗*p* < 0.001). *E*, HEK293 cells were transfected for 48 h with plasmid encoding V5-Tau, HA-DYRK1A, or Myc-PPM1B alone or in combination. Cells were lysed in either oligomerization buffer containing Triton X-100 or lysis buffer containing 0.5% Nonidet P-40. The proper expression of transiently transfected proteins in cell lysates was identified with immunoblotting with anti-V5 (Insoluble or Inputs), anti-HA, or anti-Myc antibodies (Inputs). *F*, all data represent the mean ± SD of three independent experiments (∗∗*p* < 0.01, ∗∗∗*p* < 0.001).

Excessive phosphorylation of tau protein can result in the self-assembly of oligomers and aggregation into paired helical filaments, which are involved in the pathogenesis of AD, frontotemporal dementia, and other tauopathies (21). Lastly, we examined the effect of PPM1B-mediated DYRK1A dephosphorylation on tau pathology. To determine whether PPM1B regulates tau dimerization and its further oligomerization, HEK293 cells were transfected with plasmids encoding V5-tagged tau, GFP- tagged tau, HA-tagged DYRK1A, or Myc-PPM1B alone or in combination. Cell lysates were then immunoprecipitated with anti-V5 antibody, followed by immunoblotting with anti-GFP antibody. This analysis allows us to assess the binding of two different forms of tagged tau, which reflects tau oligomerization. As shown in Figure 6, *A* and *B*, there was no interaction between V5-tau and GFP-tau in the absence of DYRK1A or PPM1B. Overexpression of DYRK1A markedly induced the binding of V5-tau to GFP-tau. These data suggest that DYRK1A stimulates tau oligomerization possibly *via* tau phosphorylation, which is consistent with the previous finding (22). Moreover, PPM1B suppressed this stimulatory effect of DYRK1A, reducing the interaction between V5-tau and GFP-tau by more than 90% (Fig. 6, *A* and *B*).

Similarly, the results of filter trap assay revealed that DYRK1A increased the amount of 1% SDS-insoluble tau aggregates by approximately twofold (Fig. 6, *C* and *D*). However, the presence of PPM1B reduced DYRK1A-induced tau aggregation to the control level (Fig. 6, *C* and *D*).

The effect of PPM1B on the formation of tau aggregates was further examined by separating the Triton X-100-insoluble fraction from the cell lysates, followed by immunoblotting of the insoluble and soluble samples with anti-V5 (tau-tagging) antibody. As shown in Figure 6, *E* and *F*, expression of V5-tau alone produced a significant amount of Triton X-100-insoluble tau aggregates. Similar to other results, DYRK1A also promoted the level of tau aggregates by 1.8-fold, which was then rescued and restored to the basal level by PPM1B (Fig. 6, *E* and *F*).

The overall results suggest that PPM1B decreases tau phosphorylation and oligomerization through the reduction of DYRK1A activity.

### PPM1B inhibits toxic tau aggregation and reduces cellular toxicity under conditions of serum deprivation in stable DYRK1A-overexpressing H19-7 cells

**Figure 7:**
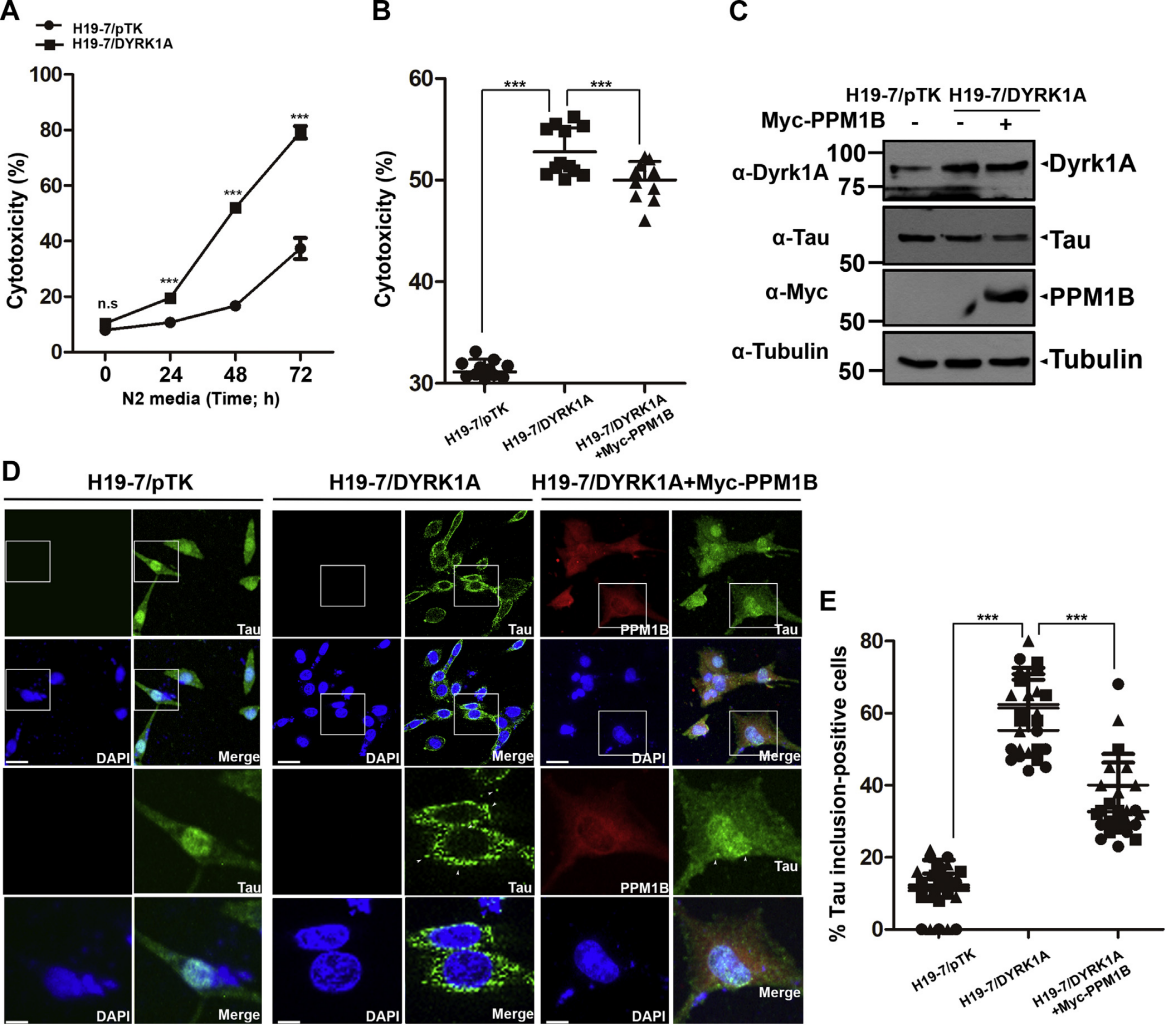
**PPM1B reduces serum-deprivation-induced cytotoxicity in H19-7 cell stably overexpressing DYRK1A through inhibition of toxic tau aggregate formation.***A*, H19-7 cells stably overexpressing DYRK1A (H19-7/DYRK1A) or control H19-7/pTK cells were incubated in N2 medium for 0 to 72 h, as indicated. Cytotoxicity was measured by assay of LDH release into the supernatant. The graph represents the mean of six independent experiments ± SD (∗∗∗*p* < 0.001). *B*‒*D*, the H19-7/DYRK1A and H19-7/pTK cells were either mock-transfected or transfected for 24 h with plasmid encoding Myc-PPM1B and then incubated in fresh N2 medium for 48 h. *B*, cytotoxicity was measured by LDH release into supernatant. The graph represents the mean of 12 independent experiments ±SD (∗∗∗*p* < 0.001). *C*, cell lysates were analyzed by western blotting with the indicated antibodies. The proper expression of stably and transiently transfected proteins in cell lysates was identified with immunoblot analysis using anti-DYRK1A, anti-tau, or anti-Myc antibodies, respectively. Tubulin served as a loading control. *D*, cells were washed with PBS, fixed with 3.7% formaldehyde, permeabilized with 0.2% Triton-X-100 in PBS, blocked with 1% BSA in PBS, and then stained with anti-Tau (*green*) and anti-Myc antibody (*red*). Nuclei were counterstained with DAPI (*blue*). Representative confocal images of immunostained cells are shown with the low and high magnification. The *bottom panels* showed enlarged views of the *white-boxed area* in the *upper panels*. Scale bar: 20 μm (the *upper panel*) and 10 μm (the *lower panel*). The *arrowheads* indicate tau aggregation dots. *E*, the percentage of tau-inclusion-positive cells was calculated by dividing the number of cells showing one or more intracellular tau inclusions by total number of tau-stained cells. Tau-inclusion-positive cells were chosen as the cells containing aggregated tau-foci, and the percentage of cells with foci was determined by visual inspection of at randomly chosen fixed areas (n = 9), which have approximately 5 to 10 cells. The three different symbols in the graph (*i.e.*, *filled circles*, *squares*, and *triangles*) represented the data obtained from three independent experiments, respectively. The graph values are expressed as mean ± SD and represents at least three independent experiments (∗∗∗*p* < 0.001).

We have previously reported that overexpression of DYRK1A in hippocampal progenitor H19-7 (H19-7/DYRK1A) cells caused an increase in tau phosphorylation and inclusion formation (23). Furthermore, serum deprivation of H19-7/DYRK1A cells caused a much greater increase in apoptotic cell death than in the control H19-7/pTK cells (23). Consistent with the previous finding, the LDH assay confirmed that H19-7/DYRK1A cells showed a much larger increase in cytotoxicity under serum deprivation than the control H19-7/pTK cells (Fig. 7*A*). Next, we examined whether the presence of PPM1B affects serum deprivation-induced cell death in H19-7/DYRK1A cells. As shown in Figure 7*B*, the presence of PPM1B considerably reduced the cytotoxicity of H19-7/DYRK1A under serum deprivation. These results might be explained by the previous and present finding that PPM1B dephosphorylates pDYRK1A at S258 and downregulates DYRK1A kinase activity, consequently decreasing the toxic modification of two AD-pathogenic proteins, such as tau and APP (23). To validate this hypothesis, we also measured the change in tau inclusions. Confocal microscopic analysis revealed that control H19-7/pTK cells do not have a significant level of intracellular tau inclusion. However, compared with control cells, there was a fivefold increase in tau inclusion formation in H19-7/DYRK1A cells. Interestingly, overexpression of Myc-PPM1B in H19-7/DYRK1A cells suppressed tau aggregation by more than 40% (Fig. 7, *C*–*E*).

Taken together, these data demonstrated that PPM1B inhibits serum deprivation-induced toxic tau aggregation and reduces cellular toxicity in stable DYRK1A-overexpressing H19-7 cells.

## Discussion

Concerning the regulation of DYRK1A *via* its intramolecular interaction, three amino acids have been documented for autophosphorylation—S97, Y321, and S529 (24). All DYRK family members are characterized by a conserved Tyr-X-Tyr motif in the activation loop of the catalytic domain (25), and phosphorylation of the second Tyr residue of this motif is necessary for kinase activity (26). In DYRK1A, Y321 corresponds to this site and becomes autophosphorylated. In order to phosphorylate substrates, DYRK1A must be activated and activation is accomplished by intramolecular autophosphorylation at the conserved Tyr321 residue in its activation loop during protein translation (5). A conserved Tyr-X-Tyr motif (Tyr319-Tyr321 in DYRK1A) in a surface loop is located between the conserved subdomains VII and VIII of the catalytic domain, and substitution of Tyr321, but not of Tyr319, with phenylalanine clearly suppresses the enzyme activity (26). DYRK1A is thought to adopt an active conformation similar to the known structures of phosphorylated ERK and p38γ (27).

DYRK1A was also reported to autophosphorylate Ser529 in the C-terminal PEST domain, which triggers a conformational change in DYRK1A, promotes the protein interaction of DYRK1A with 14-3-3β, but has no effect on intrinsic kinase activity (18). The C-terminal region of the DYRK1A catalytic domain contains an uncharged hydrophilic amino acid, and this region directly follows the kinase domain (28). Furthermore, the PEST region is located on the C-terminal portion of DYRK1A, which also harbors a repeat of 13-histidines. The binding of DYRK1A to 14-3-3β requires the phosphorylation of Ser529. Phosphorylation of Ser529 appears not to interfere with Tyr321 autophosphorylation, suggesting that autophosphorylation of Tyr321 in the activation loop is an earlier event (18). The present study verified that the phosphorylation of DYRK1A at S529 is required for the interaction with 14-3-3β. In addition, the finding that DYRK1A-S529A mutant displays comparable kinase activity confirmed that DYRK1A activity is unaffected by S529 phosphorylation.

Recently, Ser97 residue was demonstrated to autophosphorylate, stabilizing DYRK1A by blocking its degradation, but not affecting the catalytic activity (24). The Ser97 autophosphorylation is a one-time intramolecular process during the translation or folding process because it is not catalyzed by mature DYRK1A (24). A folding intermediate of DYRK1A autophosphorylates Tyr319 and Tyr321 after Ser97 in an intermolecular manner, subsequently preventing degradation of DYRK1A.

Along with these three sites, we recognized another novel residue that becomes autophosphorylated in DYRK1A. To identify this residue, Ser258, *in vitro* kinase assays were employed with serial deletion mutants of DYRK1A containing or lacking the kinase domain, to distinguish between phosphorylation of DYRK1A peptides from the kinase activity of DYRK1A or other source. Similar to Y321, it was found that autophosphorylation of S258 is necessary for DYRK1A activity. This action may be possible through the conformational change in DYRK1A after modification, by providing a more suitable environment for the kinase reaction, substrate binding, and/or the more efficient release of product. However, for the *in vitro* kinase assay, anti-DYRK1A complexes were prepared and utilized as the source of DYRK1A. Although the LC-MS/MS analysis failed to find a kinase bound to DYRK1A in HEK293 cells, it did not completely exclude the possibility that this complex might have a minor-contaminating, but previously unknown kinase that binds the N-terminal 159 to 317 amino acid region of DYRK1A stably and contributes the considerable phosphorylation of DYRK1A-S258. Therefore, more thorough and organized sets of experiment would be required to further examine the mechanism underlying activation of DYRK1A upon S258 phosphorylation.

**Figure 8:**
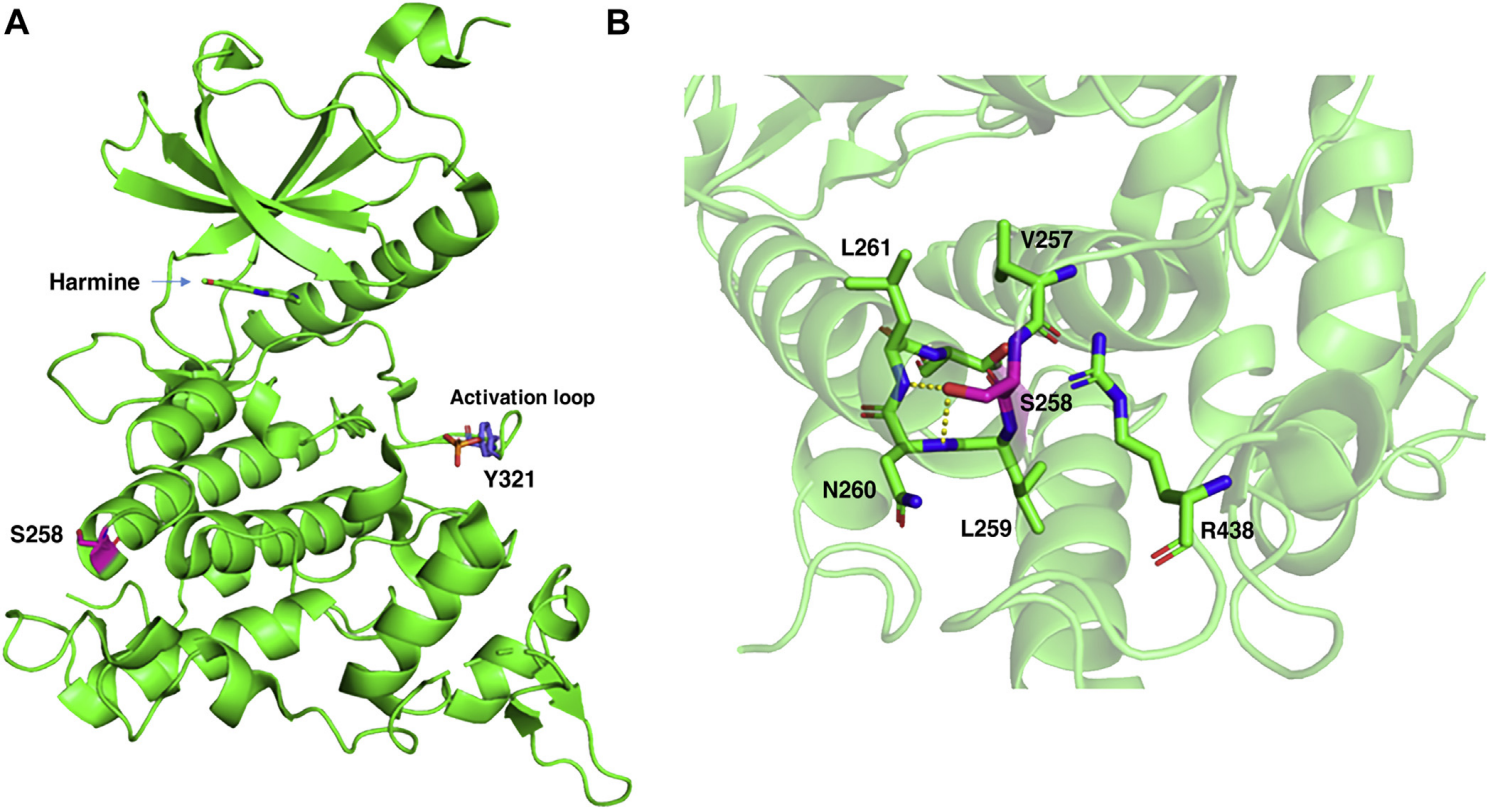
**Structural analysis of the catalytic site and the surrounding region in human DYRK1A.***A* and *B*, structural analysis of the catalytic site and the surrounding region, including residue S258 (*A*), and a higher-magnification image of the area around S258, lined with several neighboring amino acids (*B*) in the human DYRK1A–harmine complex from the RCSB protein database (https://doi.org/10.2210/pdb3ANR/pdb) are shown.

Structural analysis of the human DYRK1A–harmine complex from the RCSB protein database (https://doi.org/10.2210/pdb3ANR/pdb) revealed that residue Y321 is not located inside or in close proximity to the active site of DYRK1A, which strongly binds to the β-carboline alkaloid harmine, the potent and specific inhibitor of DYRK1A (Fig. 8). However, Y321 is located in the activation loop that protrudes from the active site. As Y321 is directly associated with the active site *via* this link, autophosphorylation of Y321 is able to affect the catalytic action in some way *via* a change in the activation loop (Fig. 8). Like Y321, the DYRK1A-S258 residue is located at the surface of the protein and appears to be suitable for the modification, including phosphorylation. In addition, S258 is not located in or not closely associated with the active site of DYRK1A. Nevertheless, there is not an activation loop around the S258 residue, which is different from Y321. The right panel of Figure 8 indicated that S258 forms hydrogen bonds with the amine group of the peptide bond from the neighboring N260 and L261 residues. Therefore, the phosphorylation of S258 might trigger a conformational change near these bonds, subsequently and indirectly affecting the distant active site of DYRK1A and its enzymatic action.

Regarding the substrates of PPM1B, there have been several targets reported to date. For example, PPM1B dephosphorylates IKKβ, TBK1, and SIRT2 (29). The first two targets are negatively regulated by PPM1B, whereas the last substrate is positively regulated by PPM1B (30). For example, treatment of HEK293-T cells with TNF-α promotes the phosphorylation of IKKβ at S177 and S181, stimulating IKKβ activity and the downstream NF-κB signaling pathway (13). In addition to these molecules, we show here that DYRK1A is a novel substrate of PPM1B, which negatively regulates DYRK1A activity.

Whereas PPM1A and PPM1B share 76% amino acid sequence identity (31, 32), their protein structures are known to be very similar. Interestingly, we showed that both PPM1A and PPM1B bind to DYRK1A. However, PPM1B, but not PPM1A, inhibits DYRK1A kinase activity and reduces tau phosphorylation. These results suggest that although PPM1A and PPM1B have very similar structures, their physiological functions might be distinct. These differential effects may be conveyed through different targets. The current study additionally revealed DYRK1A as a novel substrate of PPM1B.

In addition to tau, DYRK1A directly modifies other NDD-associated targets, including APP, presenilin-1, α-synuclein, and parkin, affecting the formation of toxic amyloid inclusions and the pathogenesis of AD and PD (33). Therefore, inhibition of DYRK1A by PPM1B may or may not also modulate these multiple factors in concert, affecting the formation of Aβ-containing senile plaques or α-synuclein-containing Lewy bodies and the progression of AD and PD. Moreover, DYRK1A phosphorylates many nuclear and cytosolic substrate proteins at serine or threonine residues (9), including transcription factors (CREB, NF-AT, STAT3, FOXO1, and Gli-1), splicing factors (cyclin L2, SF2, and SF3), translation factor (eIF2Be), synaptic proteins (dynamin I, amphiphysin I, and synaptojanin I), and miscellaneous proteins (glycogen synthase, caspase-9, and Notch). These data suggest that PPM1B may also affect many of these physiological processes *via* DYRK1A regulation. So, it would be interesting to further examine these aspects. Supporting this idea, previous studies employing chemical DYRK1A inhibitors have demonstrated a reduction in amyloid pathology, insoluble tau phosphorylation, and neuroprotection in mice (34, 35, 36). For example, a novel, potent, and selective oral DYRK1A inhibitor, SM07883, significantly reduced the deleterious effects of pathological tau and associated neuroinflammation in transgenic DYRK1A mice (37, 38).

**Figure 9:**
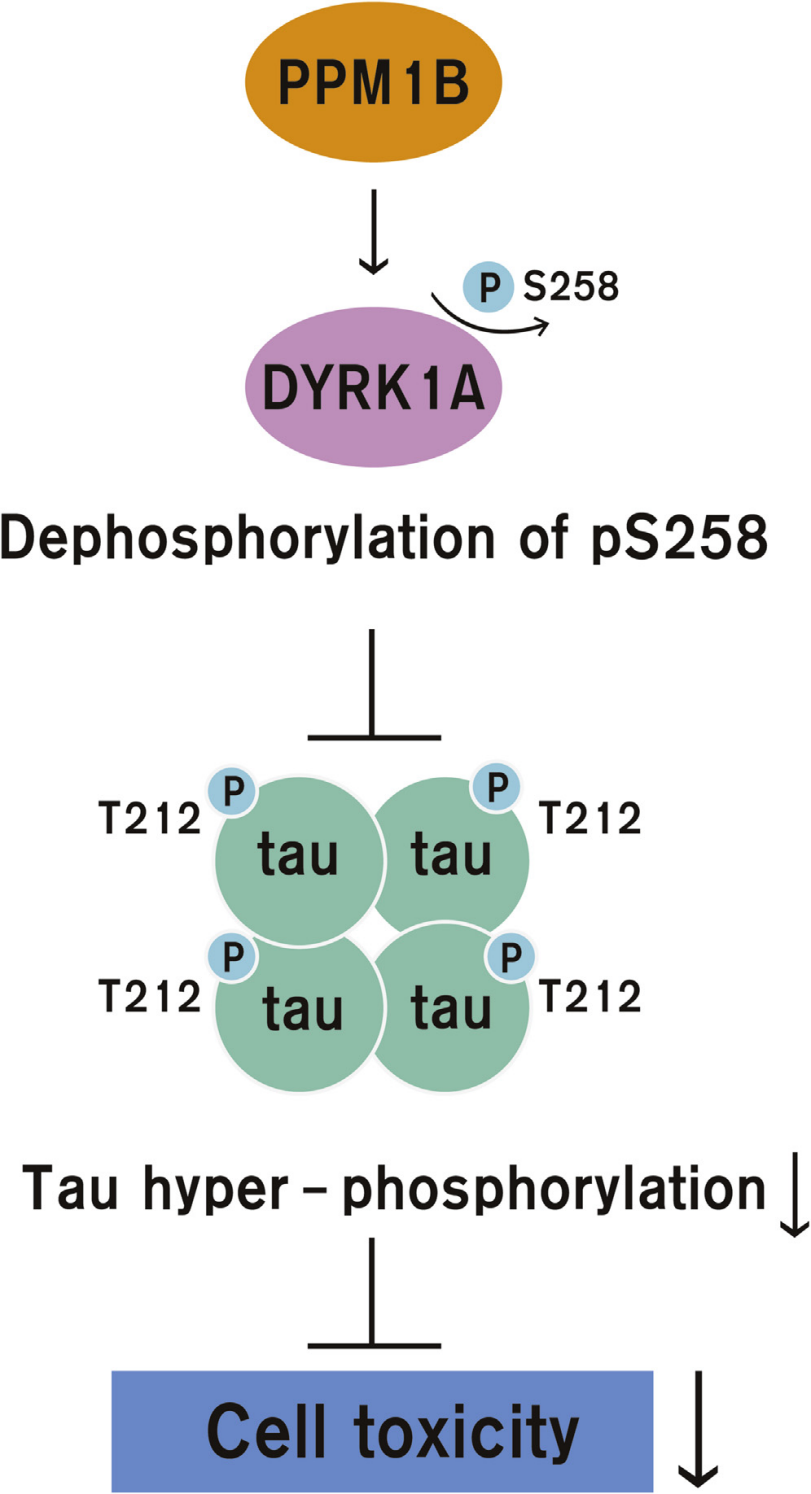
**Schematic diagram for the role of PPM1B to regulate DYRK1A.** PPM1B binds to DYRK1A and dephosphorylates its Ser258 residue, which was identified as the novel autophosphorylation site of Dyrk1A. Consequently, PPM1B decreases the kinase activity of DYRK1A, causing reduction of toxic tau phosphorylation at Thr212 residue and its neuronal toxicity in hippocampal progenitor H19-7 cells.

Our study demonstrates that PPM1B directly dephosphorylates DYRK1A at S258 and negatively regulates its kinase activity (Fig. 9). Consequently, PPM1B-mediated dephosphorylation of DYRK1A leads to a decrease in tau phosphorylation and toxic tau aggregation. These data suggest that PPM1B, as an upstream modulator of DYRK1A affecting toxic tau pathology, is a potential novel therapeutic target for AD pathology seen in AD and/or DS.

## Experimental procedures

### Materials

Dulbecco's modified Eagle medium (DMEM), fetal bovine serum (FBS), Alexa Fluor 488-conjugated mouse IgG, Alexa Fluor 546-conjugated rabbit IgG, ProLong Gold Antifade Mountant with 4′,6-diamidino-2-phenylindole (DAPI), rabbit polyclonal anti-p-tau (Thr212) antibodies (cat. #: 44-740G), mouse monoclonal anti-Xpress (R910-25), and anti-V5 (R960-25) antiserum were purchased from Invitrogen. Protein A-Sepharose beads and Ni-NTA agarose beads were purchased from GE Healthcare Life Sciences. Enhanced chemiluminescence (ECL) reagents were purchased from AbClon. Mouse monoclonal anti-FLAG (cat. #: F3165-1MG) and polyethylenimine (PEI) were purchased from Sigma-Aldrich. HRP-conjugated anti-rabbit (cat. #: AP132P) and anti-mouse (AP124P) secondary antibodies were purchased from EMD Millipore. Mouse anti-pTyr (cat. #: 05-321) and anti-pSer (05-1000) antibodies were purchased from Millipore. Rabbit anti-pThr antibodies (cat. #: ab9337) were purchased from Abcam. Monoclonal mouse anti-DYRK1A (cat. #: PAB10345) and anti-Myc (H00001859-M01) antibodies were purchased from Abnova. Rabbit polyclonal anti-FLAG (cat. #: F7425-2MG), polyclonal anti-Myc (cat. #: sc-789), anti-actin (sc-47778), anti-Hsp90 (sc-13119), mouse anti-GFP (sc-9996), and anti-tubulin (sc-5286) antibodies were purchased from Santa Cruz Biotechnology. Polyclonal anti-HA (cat. #: PAB0801) and monoclonal anti-PPM1B (H00005495-M01) IgGs were obtained from Abnova. Monoclonal anti-HA (cat. #: 901502) and rabbit polyclonal anti-V5 (cat. #: ab9116) antibodies were purchased from Covance and Abcam, respectively. Alexa Fluor 488 conjugated anti-mouse (cat. #: A-11029) and Alexa Fluor 594 conjugated anti-rabbit (A-11012) secondary antibodies were purchased from Invitrogen.

### DNA constructs

Mammalian constructs encoding rat wild-type Dyrk1a N-terminally tagged with Xpress- and 6 × His (pXpress-His6-DYRK1A-WT), its kinase-inactive mutant having the substitution of K188R (pXpress-His6-DYRK1A-KD), and FLAG-tagged wild-type Dyrk1a (pRK5-FLAG-DYRK1A) were prepared as described previously (39). Plasmids encoding Myc-tagged wild-type DYRK1A, HA-tagged wild-type DYRK1A, and various Xpress-tagged DYRK1A deletion mutants, such as DYRK1A^159–763^, DYRK1A^317–763^, or DYRK1A^417–763^, were prepared as described previously (40). Plasmids encoding human 383-amino acid isoform of tau (0N4R form) in pCMV-V5 and pGFP were kindly provided by Y.K. Jung (Seoul National University, Seoul, Korea). Bacterial plasmids encoding His-fused tau were generated by PCR amplification using the forward primer 5′-GTGGACAGCAAATGGGTCGCGATGGCTA GCCCCGCCAGGAG-3′ and the reverse primer 5′-GTGGTGGTGGTGCTCGAGTGCCAAAC CCTGCTTGGCCAGGGA-3′. Tau product was then digested with *BamH*1 and *Not*1 and inserted into vector pET28a-His. Human cDNAs encoding wild-type PPM1B and its phosphatase-inactive mutant (R179G; PPM1B-MT) were inserted into vector pcDNA3.1-Myc or pET28a-His. Bacterial plasmid encoding His-fused PPM1B was amplified by PCR using the forward primer 5′-GAAGAATTCATGGGTGCATTTTT GGATAAACCC-3′ and the reverse primer 5′-GAAGCGGCCGCTATTTTTTCACCACTCATCTTTGTCCC-3′, and the product was digested by *EcoR*1 and *Not*1. To make a variety of DYRK1A constructs encoding its deletion or point-mutants, site-directed mutagenesis was performed using the QuikChange XL site-directed mutagenesis kit (Agilent Technologies). All cDNA constructs were confirmed by DNA sequencing (COSMO Genetech).

### Cell culture and DNA transfection

Rat immortalized hippocampal progenitor H19-7 cells, human dopaminergic neuroblastoma SH-SY5Y cells, and human embryonic kidney 293 (HEK293) cells were maintained in DMEM containing 10% FBS and 100 U/ml penicillin-streptomycin. Cells were grown at 37 °C in 5% CO_2_. All DNA transfections were performed using PEI and Lipofectamine 2000 reagents, according to the manufacturer’s protocol. The H19-7 cells stably overexpressing DYRK1A (H19-7/DYRK1A) and its control cell line carrying vector pTK-Hyg (H19-7/pTK) were prepared as described previously (23).

### In-gel trypsin digestion and protein identification by LC-MS/MS

HEK293 cells were cotransfected with pTK-Hyg and pcDNA3.1-FLAG-DYRK1A and screened with 250 μg/ml of hygromycin B. To detect levels of DYRK1A in each cell line, 75 μg of each cell lysate was analyzed by immunoblotting with anti-DYRK1A, anti-FLAG, or anti-Hsp90 antibody. To map DYRK1A binding partners from FLAG-DYRK1A-overexpressing HEK293 cells (Flag-DYRK1A), ESI-MS/MS was performed with parental HEK293 vector cells (Vector) as a control, as previously described (32). Overexpressed wild-type FLAG-DYRK1A proteins were purified using FLAG-M2 agarose (Sigma) and separated by SDS-PAGE. In-gel digestion with 25 ng/μl trypsin (Promega) and 25 ng/μl chymotrypsin (Roche) was performed simultaneously for 16 h at 37 °C. The peptide analysis was carried out using a QSTAR Pulsar quadrupole time-of-flight (TOF) mass spectrometer (Applied Biosystems) equipped with nanoelectrospray ion sources (Protana). The database search for sequenced peptides was performed in the National Center for Biotechnology Information (Bethesda, MD, USA) nonredundant database using the MASCOT software package (Matrix Sciences).

### Recombinant protein expression and purification

*E. coli* BL21 (DE3)-Codon Plus competent cells (Invitrogen) were transformed with pET28a-His-Tau plasmid. A single colony was inoculated into 10 ml selective LB broth containing 50 μg/ml kanamycin and grown overnight at 37 °C. Cultured cells (10 ml) were inoculated into 100 ml selective LB broth containing 50 μg/ml kanamycin and grown at 37 °C with gentle agitation. Cell cultures were grown until OD at 600 nm reached 0.6 and then incubated with 0.05 mM isopropyl-β-D-1-thiogalactopyranoside (IPTG) for 2 h at 25 °C, pelleted by centrifugation at 3000*g* for 15 min at 4 °C. The cells were sonicated in lysis buffer (50 mM Tris-HCl, pH 7.5, 1% NP-40, 1% Triton-X100, 10% glycerol, 150 mM NaCl, and 20 mM imidazole). After centrifuging the cell lysates at 6000*g* for 15 min at 4 °C, the supernatant was incubated with Ni-NTA agarose beads overnight at 4 °C. The beads were then washed three times with washing buffer (50 mM Tris-HCl, pH 7.5, 1% NP-40, 1% Triton-X100, 10% glycerol, 150 mM NaCl, and 100 mM imidazole), and the recombinant tau proteins were eluted by adding elution buffer containing 500 mM imidazole. Bacterial recombinant PPM1B protein was produced and purified with the same protocol.

### Immunoprecipitation and western blot analysis

Cell lysates were prepared by rinsing cells with ice-cold phosphate-buffered saline (PBS) and lysed with 1% Nonidet P-40 lysis buffer (50 mM Tris, pH 7.5, 150 mM NaCl, 1% Nonidet P-40, 10% glycerol, 0.2 mM phenylmethylsulfonyl fluoride, 1 mM Na_3_VO_4_, 10 mM NaF, and 1 × protease inhibitor cocktail [including 1 μg/ml aprotinin, 1 μg/ml leupeptin, and 1 μg/ml pepstatin]). Cells were then scraped, and supernatants were collected after centrifugation at 15,700*g* for 15 min at 4 °C. For immunoprecipitation, 1 μg of appropriate antibody was incubated with 500 μg cell lysate overnight at 4 °C with gentle rotation. The mixture was then incubated with 30 μl of a 1:1 mixture of protein A-Sepharose bead suspension for 2 h at 4 °C. Beads were pelleted by centrifugation at 9300*g* for 1 min and washed three times with 1% Nonidet P-40 lysis buffer. The immunocomplexes were dissociated by boiling in SDS-PAGE sample buffer, separated on SDS-PAGE, and transferred to nitrocellulose membranes (Millipore). Membranes were blocked for 1 h at room temperature using TRIS-buffered saline with Tween (TBST; 25 mM Tris, pH 7.5, 150 mM NaCl, and 0.1% Tween20) containing 5% nonfat dry milk and then incubated overnight at 4 °C in TBST containing the appropriate antibody. Membranes were then washed three times in TBST and incubated with secondary IgG-coupled horseradish peroxidase for 2 h. The blots were washed three times with TBST, and the bands were visualized using ECL reagents (Abclon), following the manufacturer’s instructions.

### *In vitro* kinase assay

After cell lysis in lysis buffer, approximately 1000 μg of protein was incubated with anti-Myc or anti-HA antibodies for overnight at 4 °C. The immunocomplexes were mixed with protein A-Sepharose beads for 2 h at 4 °C. Beads were centrifuged at 6000*g* for 30 s and consecutively washed with lysis buffer and with the kinase reaction buffer (20 mM HEPES, pH 7.2, 20 mM MgCl_2_, 5 mM MnCl_2_, 1 mM DTT). Complexes immunoprecipitated with Myc- or HA-tagged DYRK1A were mixed with 1 μg recombinant tau protein, the kinase reaction buffer, 0.2 mM Na_3_VO_4_, and 10 μM ATP. *In vitro* kinase reaction was initiated by the addition of 10 μCi [γ-^32^P]ATP. The reaction was allowed to proceed for 30 min at 30 °C and terminated by the addition of SDS-PAGE sample buffer. The samples were then resolved by SDS-PAGE and incorporated [γ-^32^P]ATP radioisotope was detected by autoradiography.

### *In vitro* phosphatase assay

Complexes immunoprecipitated with Myc- or HA-tagged DYRK1A were washed twice times with 1× PPM1B buffer (29 mM HEPES, pH 7.4, 20 mM MgCl_2_, 0.03% β-mercaptoethanol) and mixed with 1 μg of recombinant PPM1B-WT or PPM1B-MT protein for 30 min at 30 °C. The reactions were stopped by addition of 2× SDS sample buffer, and the mixtures were analyzed by SDS-PAGE and visualized by autoradiography.

### Filter trap and oligomerization assay for tau aggregates

HEK293 cells were transfected with pRK5-HA-DYRK1A, pcDNA3.1-Myc-PPM1B, and/or pCMV-V5-Tau for 24 or 48 h. Cells were lysed using lysis buffer (50 mM Tris, pH 7.4, 120 mM NaCl, 1 mM EDTA, 0.5% Nonidet P-40, and the protease inhibitor cocktail). After brief sonication, equal amounts of cell lysates were passed through a nitrocellulose filter with pore size of 0.2 μm. The resultant membranes were washed three times with lysis buffer containing 1% SDSand blocked in 5% nonfat milk for 1 h, followed by immunoblotting with anti-V5 antibody. For the assay of tau aggregates, cells were lysed with oligomerization buffer (20 mM Tris, pH 7.4, 150 mM NaCl, 1% Triton-X-100, and 1× protease inhibitor cocktail), and the supernatants were collected after centrifugation at 15,700*g* for 15 min at 4 °C. Cell lysates were resuspended with SDS sample buffer, resolved by SDS-PAGE, and transferred to nitrocellulose membranes.

### Analysis of serum-deprivation-induced cell death in H19-7 cells

Cellular cytotoxicity was measured using the LDH Cytotoxicity Detection Kit (Takara Korea), according to the manufacturer protocol. To assess the serum deprivation-induced cytotoxicity in H19-7 cells, N2 medium was added to the cells before reaching 70∼90% confluence, and cells were cultured at 37 °C for 0 to 72 h. The supernatant (100 μl) was then transferred to a new 96-well plate and 100 μl of LDH substrate mix was added. After 20-min incubation at room temperature, the absorbance of the sample was measured at 490∼600 nm.

### Confocal microscopic analysis of tau inclusions in H19-7 cells

H19-7/DYRK1A and control H19-7/pTK cells were seeded onto poly-L-lysine-coated coverslips in 6-well plates to approximately 50∼70% confluence at 37 °C. To induce toxic tau aggregation followed by apoptotic cell death, cells were incubated with N2 medium for 48 h at 37 °C, washed twice with PBS (pH 7.4), and immediately fixed with 3.7% formaldehyde for 30 min. After fixation, cells were permeabilized with 0.2% Triton X-100 for 30 min and blocked with 1% bovine serum albumin (BSA) for 30 min at room temperature, followed by immunostaining with anti-tau or anti-Myc antibody. FITC-conjugated secondary antibody was then used to detect the primary antibodies. The samples were counterstained with DAPI, mounted, and analyzed by LSM 880 confocal microscopy (Carl Zeiss). The data were processed by Zeiss LSM Image Browser (Carl Zeiss).

### Statistical analyses

The S-Group means of samples were compared using Student’s *t*-test and the IBM SPSS statistical analysis software (version 23.0), and *p* < 0.05 was considered statistically significant. Values are reported as mean ± SD of at least three independent experiments. The densities of western blot bands were measured by GelQuant.NET software (version 1.8.2), according to the protocol provided by the manufacturer (http://www.biochemlabsolutions.com).

## Data availability

All data that support the findings of this study are contained within the article and are available from the corresponding author upon reasonable request.

## Conflict of interest

The authors declare that they have no conflicts of interest with the contents of this article.
